# Ubiquitous Presence and Novel Diversity of Anaerobic Alkane Degraders in Cold Marine Sediments

**DOI:** 10.3389/fmicb.2015.01414

**Published:** 2015-12-17

**Authors:** Antje Gittel, Jonathan Donhauser, Hans Røy, Peter R. Girguis, Bo B. Jørgensen, Kasper U. Kjeldsen

**Affiliations:** ^1^Center for Geomicrobiology, Department of Bioscience, Aarhus University Aarhus, Denmark; ^2^Department of Organismic and Evolutionary Biology, Harvard University Cambridge, MA, USA

**Keywords:** anaerobic bacteria, gas seepage, marine sediments, (1-methyl-) alkyl succinate synthase, microbial alkane degradation, short-chain alkanes

## Abstract

Alkanes are major constituents of crude oil and are released to the marine environment by natural seepage and from anthropogenic sources. Due to their chemical inertness, their removal from anoxic marine sediments is primarily controlled by the activity of anaerobic alkane-degrading microorganisms. To facilitate comprehensive cultivation-independent surveys of the diversity and distribution of anaerobic alkane degraders, we designed novel PCR primers that cover all known diversity of the 1-methylalkyl succinate synthase gene (*masD/assA*), which catalyzes the initial activation of alkanes. We studied *masD/assA* gene diversity in pristine and seepage-impacted Danish coastal sediments, as well as in sediments and alkane-degrading enrichment cultures from the Middle Valley (MV) hydrothermal vent system in the Pacific Northwest. *MasD/assA* genes were ubiquitously present, and the primers captured the diversity of both known and previously undiscovered *masD/assA* gene diversity. Seepage sediments were dominated by a single *masD/assA* gene cluster, which is presumably indicative of a substrate-adapted community, while pristine sediments harbored a diverse range of *masD/assA* phylotypes including those present in seepage sediments. This rare biosphere of anaerobic alkane degraders will likely increase in abundance in the event of seepage or accidental oil spillage. Nanomolar concentrations of short-chain alkanes (SCA) were detected in pristine and seepage sediments. Interestingly, anaerobic alkane degraders closely related to strain BuS5, the only SCA degrader in pure culture, were found in mesophilic MV enrichments, but not in cold sediments from Danish waters. We propose that the new *masD/assA* gene lineages in these sediments represent novel phylotypes that are either fueled by naturally occurring low levels of SCA or that metabolize medium- to long-chain alkanes. Our study highlights that *masD/assA* genes are a relevant diagnostic marker to identify seepage and microseepage, e.g., during prospecting for oil and gas, and may act as an indicator of anthropogenic oil spills in marine sediments.

## Introduction

Marine ecosystems are continually exposed to hydrocarbons including alkanes, which are major constituents of crude oil and are released from both natural oil and gas seeps and anthropogenic sources (Sassen et al., 2004; Etiope and Ciccioli, 2009). Due to their chemical inertness, the fate of alkanes in the environment is primarily governed by the activities of hydrocarbon-degrading microorganisms (Head et al., 2006; Atlas and Hazen, 2011; Callaghan, 2013). At natural oil seeps, microbial populations are known to specialize in hydrocarbon degradation (Rueter et al., 1994; Teske et al., 2002; Joye et al., 2004; Kniemeyer et al., 2007; Orcutt et al., 2010; Kleindienst et al., 2014, 2015; Underwood et al., 2015) and may respond rapidly to a dramatic release of oil into the environment, e.g., from accidental oil spills, or deposition of oil-contaminated drill cuttings on the seafloor (King et al., 2015). As a consequence, the transformation and mineralization of oil constituents accelerates by orders of magnitude, as was observed following the 2010 Deepwater Horizon disaster (reviewed by Kimes et al., 2014). To date, the technical application of hydrocarbon biodegradation in bioremediation and attenuation strategies has primarily been centered around aerobic processes (Atlas and Hazen, 2011; McGenity et al., 2012; Fuentes et al., 2014). However, in marine ecosystems, especially in marine sediments, anaerobic degradation processes play a crucial role in the removal of spilled oil, black carbon, and ashes from oil burning that deposit on the seafloor (Kimes et al., 2013; Valentine et al., 2014; Chanton et al., 2015).

Similar to the anaerobic degradation of aromatic hydrocarbons, fumarate addition was established as the biochemical mechanism for n-alkane activation in the two anaerobic model organisms, *Desulfatibacillum alkenivorans* AK-01 (Callaghan et al., 2006, 2008, 2012) and *Aromatoleum* HxN1 (Wilkes et al., 2002; Grundmann et al., 2008). In fumarate addition, n-alkanes are initially activated by carbon–carbon addition of an alkyl radical to fumarate at the subterminal (Kropp et al., 2000; Rabus et al., 2001; Wilkes et al., 2002, 2003; Cravo-Laureau et al., 2005; Davidova et al., 2005) or terminal carbon (Kniemeyer et al., 2007) of the n-alkane substrate. Further degradation of the alkyl-substituted succinate metabolites proceeds via carbon-skeleton rearrangement followed by carboxylation and β-oxidation (Wilkes et al., 2002). It has been postulated that fumarate addition, i.e., the abstraction of an H atom from the alkane substrate, is catalyzed by the glycyl radical enzyme, 1-methylalkyl succinate synthase (MAS) (Grundmann et al., 2008), also named alkylsuccinate synthase (ASS) (Callaghan et al., 2008). In accordance, this enzyme is encoded in the genomes of most known anaerobic alkane degaders (Grundmann et al., 2008; Callaghan et al., 2010, 2012; Zedelius et al., 2011; Musat, 2015) and was identified in *Smithella* sp. and *Firmicutes* sp. from methanogenic alkane-degrading enrichment cultures (Tan et al., 2013; Embree et al., 2014; Tan et al., 2014a,b). MAS/ASS can be distinguished from the homologous benzyl succinate sythase (BSS), involved in the activation of aromatic hydrocarbons, and pyruvate formate-lyase (PFL) by phylogenetic analyses (von Netzer et al., 2013). Furthermore, MAS/ASS and BSS contain a single conserved cysteine residue in their active site, whereas enzymes in the PLF family typically contain two adjacent cysteines (Leuthner et al., 1998; Becker et al., 1999; Callaghan et al., 2008). Alternative activation pathways for alkane activation such as carboxylation and oxygen-independent hydroxylation have been proposed (Aeckersberg et al., 1998; So et al., 2003; Zedelius et al., 2011; Callaghan, 2013), but understanding their biochemistry is still in its infancy.

The genes involved in alkane activation by fumarate addition are currently the most relevant genetic markers for anaerobic alkane degradation and several PCR-based detection assays have been developed to target the genes that encode the α-subunit of the MAS/ASS enzyme (*masD/assA*) (Callaghan et al., 2010; Aitken et al., 2013; von Netzer et al., 2013). Using these assays, recent studies addressed the distribution and diversity of *masD/assA* genes in oil-contaminated sediments and natural hydrocarbon seeps (Callaghan et al., 2010; Acosta-González et al., 2013; Adams et al., 2013; Bose et al., 2013; Kimes et al., 2013; von Netzer et al., 2013; Kleindienst et al., 2014). However, re-evaluation of primer coverage and specificity using recent genomic and metagenomic data showed that none of these primer pairs comprehensively targeted the *masD/assA* gene diversity found in known anaerobic alkane degraders, as they excluded *Desulfothermus naphthae*^T^, *Desulfosarcina* sp. strain BuS5, and *Smithella* species (Werner, 2009; Tan et al., 2014b; Musat, 2015). Given that these primers do not recover the full diversity of *masD/assA* genes, the environmental diversity and distribution of anaerobic alkane degraders remains poorly characterized. In particular, only marginal information is available on anaerobic alkane degraders in pristine sediments, i.e., sediments that are not influenced by thermogenic oil and gas formations and are not exposed to high loads of hydrocarbons from anthropogenic sources (Acosta-González et al., 2013; Kimes et al., 2013).

The presence of short-chain alkanes (SCA, C_2_–C_5_) in the pore water of marine sediments is generally considered a result of vertical migration from subsurface accumulation of hydrocarbons or reservoirs to the sediment surface and results in the release of SCA via macroseeps (focused/diffusive flow, e.g., oil and gas seeps, mud volcanoes, pockmarks) and microseepage (diffusive flow) (Etiope, 2015). While less obvious than more charismatic sources, microseepage was estimated to represent the main contributor to the global atmospheric flux of SCA (Etiope and Ciccioli, 2009). As SCA act as greenhouse gasses and are precursors for the formation of ozone and organic aerosols (Chameides et al., 1992; Kawamura et al., 1996), the controls on microbial oxidation of SCA are of great interest in tropospheric chemistry and climate research (Musat, 2015). In the off-shore oil and gas industry, gas microseepage detection by measuring SCA concentrations has become a prospecting tool for discovering buried hydrocarbon reservoirs (Hubert and Judd, 2010). However, the applicability of this method is confounded by a lack of information on SCA concentrations in pristine marine sediments as well as on the processes that control their presence and turnover rates. In addition, considerable amounts of ethane and propane have been found in pristine marine sediments, which indicates the possiblity for biogenic SCA formation, but the distribution, magnitude and biology of such SCA-generating processes are not known (Hinrichs et al., 2006; Xie et al., 2013). Thus, developing tools to better understand the abiotic and biological factors that govern the distribution and abundance of SCA and SCA-degrading microorganisms in pristine surficial marine sediments is of environmental and ecological relevance as well as provides important information to identify microseepage sites and to interpret gas signatures in the scope of oil and gas prospecting.

The aims of this study were (1) to develop a novel *masD/assA* gene detection assay to comprehensively recover the environmental diversity of this marker gene, (2) to determine base level concentrations of SCA in pristine sediments and (3) with *masD/assA* as the proxy, to compare the phylogenetic diversity and community composition of anaerobic alkane-degrading microorganisms in seepage-impacted and pristine marine surficial sediments. In pristine, marine pelagic environments, aerobic hydrocarbon degraders are typically present at low abundance (Harayama et al., 2004). In analogy, we hypothesized that pristine marine sediments contain a rare biosphere of anaerobic alkane degraders that can provide a seed bank, which will determine the community response when exposed to hydrocarbons. We argue that the same *masD/assA* gene phylotypes are present in seepage and pristine sediments, but that certain members of the anaerobic alkane-degrading community are enriched in seepage sediments. Thus, *masD/assA* genes would represent a relevant diagnostic marker to monitor traces of accidental oil spills in marine sediments, to detect the leakage of oil pipelines and to identify microseepage during prospecting for oil and gas.

## Materials and Methods

### Sediment Sample Collection and Processing

Samples were collected from pristine cold marine sediments as well as from cold and hot sediments impacted by hydrocarbon seepage (**Table 1**). Specifically, samples from cold impacted sediments were collected in the Skagerrak (station SKA3; 57° 54.20′ N, 9°20.62′ E) and in a seepage area in Limfjorden (LF, 56° 47.88′ N, 8°31.51′ E) that was covered under hydrocarbon exploration license 3/07 (Danish Energy Agency, 2007). These gas seepage areas were identified by acoustic blanking during multibeam and sub-bottom echo sounding. Pristine sites included stations SKA1 (58° 06.21′ N, 9° 49.34′ E) and SKA4 (57° 48.40′ N, 11° 03.19′ E) in the Skagerrak, station SKA5 (55° 00.26′ N, 10° 06.48′ E) in the Lille Belt, stations M1 (56° 07.07′ N, 10° 20.79′ E) and M5 (56° 06.20′ N and 10° 27.47′ E) in Aarhus Bay and a reference site in Limfjorden (BB, 56° 50.38′ N, 9°09.32′ E). Pristine sediments were defined as sediments that are isolated from deep oil and gas formations and have not been exposed to high loads of hydrocarbons from anthropogenic sources in recent times (e.g., by exploratory drilling or oil spills). Sediment cores were collected by either Rumohr coring [1 m long, 10 cm diameter polycarbonate (PC) barrel] or gravity coring (6 m steel barrel, 12 cm diameter inner core liner) and sectioned in 10 cm depth intervals for biogeochemical and molecular biological analyses. Five cm^3^ of each section were taken with sterile cut-off syringes and stored at -80°C for subsequent molecular analysis. Sediment cores from an intertidal/shallow subtidal gas seepage site at the northern Kattegat coast (Bangsbostrand, BS, 57°23.10′ N, 10°30.92′ E) were collected manually in translucent PC tubes and kept at 4°C during transportation to the laboratory (∼2 h). Samples for molecular analysis were sampled at 5 cm depth intervals using 2.5 mL cut-off sterile syringes and subsequently stored at -80°C. The biogeochemistry of the BS site was previously described (Jensen et al., 1992; Dando et al., 1994).

**Table 1:** Sample overview, amplification results and sequencing statistics for *masD/assA* gene clone libraries from pristine and seepage-impacted environmental samples and enrichment cultures.

Sample	Sample type, location, coring method^a^	Sediment depth (cm)	Amplification result^b^	Number of clones^c^	Number of OTUs^d^	Good’s coverage^d^
**Pristine sediments**						
M1	Marine sediment, Aarhus Bay, RC	0–50	++ (45)	68	22	0.88
SKA1	Marine sediment, Skagerrak, GC	0–46	++ (55)	70	17	0.92
SKA4	Marine sediment, Skagerrak, GC	13–53	++ (55)	59	15	0.86
SKA51^e^	Marine sediment, Lille Belt, GC	3–50	++ (45)	80	22	0.9
SKA52^e^	Marine sediment, Lille Belt, GC	70–105	++ (45)	57	18	0.84
M5^f^	Marine sediment, Aarhus Bay, RC	4–52	n.a.			
BB^f^	Fjord sediment, Limfjorden, RC	0–40	n.a.			
**Seepage-impacted sediments**						
LF1^f,g^	Eutrophic fjord sediment, Limfjorden, RC	0–60	n.a.			
LF2^g^	Eutrophic fjord sediment, Limfjorden, RC	0–40	++ (45)	51	17	0.9
LF3^g^	Eutrophic fjord sediment, Limfjorden, RC	0–50	++ (45)	78	27	0.83
LF4^f,g^	Eutrophic fjord sediment, Limfjorden, RC	0–60	n.a.			
LF5^g^	Eutrophic fjord sediment, Limfjorden, RC	0–50	++ (45)	80	15	0.95
LF6^g^	Eutrophic fjord sediment, Limfjorden, RC	0–70	++ (45)	79	18	0.96
SKA3	Marine sediment, Skagerrak, RC	0–45	++ (45)	69	13	0.96
BS	Coastal sandy sediment, Bangsbostrand, PC	0–10	+ (55)	63	13	0.92
MV	Marine sediment, Middle Valley (Juan de Fuca Ridge)	0–30	+ (55)	11	4	0.82
**Enrichments**						
MV25C3	Propane-degrading enrichment, 25°C	–	++ (55)	16	1	1
MV25C4	Butane-degrading enrichment, 25°C	–	+ (45)	24	1	1
MV55C3	Propane-degrading enrichment, 55°C	–	–	–	–	–
MV55C4	Butane-degrading enrichment, 55°C	–	–	–	–	–

*^a^Coring method: Rumohr coring (RC), gravity coring (GC), or manually pushed cores (PC).*

*^b^In parentheses, number of cycles needed for successful amplification.*

*^c^Number of clones that resulted in sequences >800 nucleotides after quality check and removing the vector sequence.*

*^d^The number of OTUs and coverage were defined at a cutoff of 5% amino acid sequence difference.*

*^e^Two different depth intervals (SKA51, SKA52) were analyzed at station SKA5.*

*^f^Samples from stations M5, BB, LF1, and LF4 were sampled for hydrocarbon gas analyses only.*

*^g^Replicate sediment cores (LF1-6) were taken in a seepage area in Limfjorden and analyzed separately for hydrocarbon gasses (2) and molecular signatures (4).*

*++, strong amplicon with the correct product size. +, weak amplicon with the correct product size. -, no amplicon obtained at a maximum of 55 cycles. n.a., not analyzed.*

Samples were also collected from hydrothermal, metalliferous sediments recovered from the Chowder Hill site at the Middle Valley (MV) hydrothermal vent field, located at 2413 m water depth along the Juan de Fuca Ridge (48° 27.44′ N, 128° 42.51′ W). Sediments were collected using 7.5 cm diameter by 30 cm long PC “pushcores” using the human occupied submersible *Alvin*, during an expedition on board the R/V Atlantis in July 2010. Sample collection and processing as well as the set-up of propane- and butane-degrading MV enrichment cultures were described previously (Adams et al., 2013). The MV vent system is rich in dissolved hydrocarbon species (several 100’s of micromolar of total C_2_–C_4_ alkanes) and reduced compounds, but generally depleted in organic carbon (Cruse and Seewald, 2006; Wankel et al., 2012). The geochemistry at MV favors the enrichment of anaerobic hydrocarbon degraders and we thus used MV sediments and enrichments as references for a heavily hydrocarbon-impacted natural environment.

### Methane and Non-methane Hydrocarbon Gas Concentrations and Carbon Isotopic Composition

For hydrocarbon gas analysis, two cm^3^ of sediment was transferred into gas-tight headspace vials containing 4 mL H_2_O and 2.5 g of NaCl. The vials were crimp-sealed and stored upside down until further analysis. Gas concentrations were determined from headspace measurements using an SRI 310C gas chromatograph (methane) and an Agilent Technologies 7820A gas chromatograph (ethane, propane, butane) both equipped with flame ionization detectors. Stable carbon isotopic compositions of methane (δ^13^CH_4_) were determined by isotope ratio mass spectrometry with a pre-concentration unit (PreCon, Thermo Scientific) connected to a Delta V plus isotope ratio mass spectrometer (Thermo Scientific). Results were referenced against Vienna Pee-Dee Belemnite and expressed as: δ^13^C = ([^13^C/^12^C]_sample_/[^13^C/^12^C]_standard_ - 1) × 1000‰.

### Pure Cultures

Genomic DNA from *Desulfatibacillum alkenivorans* PF2803^T^ (DSM16129) and *Desulfatibacillum aliphaticivorans* CV2803^T^ (DSM15576) and pure cultures of *Desulfothermus naphthae* TD3^T^ (DSM13418), *Desulfoglaeba alkanexedens* ALDC^T^ (DSM18185) and *Desulfosarcina variabilis* (DSM2060^T^) were obtained from the Deutsche Sammlung von Mikroorganismen und Zellkulturen (Braunschweig, Germany). Cell pellets of *Desulfosarcina* sp. strain BuS5 were kindly provided by Florin Musat (Umweltforschungszentrum Leipzig, Germany).

### DNA Extraction and Quantification

Genomic DNA from pelleted pure cultures as well as from MV sediment samples and enrichments was extracted using phenol-chloroform (Dojka et al., 1998; Adams et al., 2013) with slight modifications to the published protocols. Prior to DNA extraction, MV samples and enrichments (0.5 g or 0.5 cm^3^, respectively) were washed with cold 100% ethanol and 0.6 N HCl to eliminate potential PCR inhibitors (metals, sulfide). Poly adenylic acid (poly A, ∼0.5 mg/mL final concentration) and pyrophosphate (0.5% final concentration) were added during the lysis step to prevent nucleic acid loss and eliminate potential PCR inhibitors (Webster et al., 2003). Incubation with lysozyme (5 mg/mL final concentration) and proteinase K (∼2 mg/mL final concentration) was followed by five freeze-thaw cycles with sodium dodecyl sulfate (SDS, ∼5% final concentration), addition of hot phenol and extraction with phenol-chloroform. Glycogen (1 μL, Roche, Basel, Switzerland) was added to assist precipitation (Paulin et al., 2013) and precipitated DNA was dissolved in molecular biology grade water (Mo Bio Laboratories, Carlsbad, CA, USA).

Genomic DNA from sediment samples (other than MV) was extracted as described previously (Kjeldsen et al., 2007) following a protocol that combined bead-beating, enzymatic and chemical lysis, and the FastDNA spin kit for soil (MP-Biomedicals, Santa Barbara, CA, USA).

DNA concentrations were measured using the Quant-iT double-stranded-DNA HS assay kit and a Qubit fluorometer (Invitrogen, Carlsbad, CA, USA).

### Primer Design and Validation

All 1-methylalkyl succinate synthase (*masD/assA*), benzyl succinate synthase (*bssA*) and napthyl-2-methyl succinate synthase (*nmsA*) gene sequences (624 total sequences) available in Genbank were retrieved and aligned in ARB (Ludwig et al., 2004) based on their translated amino acid sequences. Primers for *masD/assA* gene detection were manually designed from conserved gene regions of *Azoarcus* sp. HxN1 (AM748709), *Aromatoleum* sp. OcN1 (FN675935), *Desulfatibacillum alkenivorans* AK-01 (DQ826035 and DQ826036), *Desulfatibacillum alkenivorans* PF2803^T^ (LN868322, this study), *Desulfatibacillum aliphaticivorans* CV2803^T^ (LN868321, this study), *Desulfoglaeba alkanexedens* ALDC^T^ (GU453656), *Desulfothermus naphthae* TD3^T^ (unpublished; kindly provided by Fritz Widdel, Max Planck Institute for Marine Microbiology, Bremen, Germany), and *Desulfosarcina* sp. strain BuS5 (WP_027352796). *In silico* coverage and specificity of the primers were tested using the probe match function of ARB matching the primer sequences against the custom-made *masD/assA/bssA/nmsA* sequence database and by BLASTN searches against the NCBI nucleotide collection database. Experimental confirmation was obtained by PCR amplification of *masD/assA* genes from genomic DNA of pure cultures (**Table 2**). *Desulfosarcina variabilis* (DSM2060^T^) was used as a negative control.

**Table 2:** Experimentally tested performance of different *masD/assA* gene-targeted primers in selected pure cultures.

Culture	Aliphatic hydrocarbon substrates	Amplification with primer set^a^			
		masD1076a	masD1156a	masD1076b	masD1156b
*Desulfatibacillum alkenivorans* PF2803^T^	C8–C23 alkenes^b^	++	++	m	m
*Desulfatibacillum aliphaticivorans* CV2803^T^	C13–C18 alkanes	++	++	m	m
*Desulfothermus naphthae* TD3^T^	C6–C16 alkanes	n.t.	++	n. t.	n. t.
*Desulfoglaeba alkanexedens* ALDC^T^	C6–C12 alkanes	n.t.	++	n. t.	n. t.
*Desulfosarcina* sp. strain BuS5	C3–C4 alkanes	+	++	n. t.	m
*Desulfosarcina variabilis* ^T^	None	–	–	–	–

*^a^Primer pairs, masD1076a: 1076F/2004Rmod, masD1156a: 1156F/2004Rmod, masD1076b: 1076F/2476R, masD1156b: 1156F/2476R. Primer sequences, 1076F: 5′-TBGGHGARTWYHTGATYMG-3′, 1156F: 5′-GGHMCVTDBGTVTGGAC-3′, 2004Rmod: 5′-RTCRTCRTTDCCCCAYTTNGG-3′, 2476R: 5′-ANCCNGMNAYVCKNACRATVA-3′.*

*^b^Growth of Desulfatibacillum alkenivorans PF2803 on C5–C20 alkanes was tested, but no growth was observed (Cravo-Laureau et al., 2004).*

*++, strong amplicon of the correct product size. +, weak amplicon at low annealing temperatures (≤ 50°C). -, no amplicon. m, multiple bands including amplicons of the correct size. n. t., not tested.*

### PCR Amplification of *masD/assA* Genes from Environmental Samples

*MasD/assA* gene fragments from environmental samples were amplified via gradient PCR with the newly developed primer set masD1156a (masD1156F/masD2004Rmod; **Table 2**) and applying a temperature gradient from 49°C to 54°C. The thermal cycling conditions were as follows: 15 min at 95°C, 45–55 cycles of 30 s at 95°C, 30 s at 49–54°C, and 30 s at 72°C, completed by a final elongation step of 10 min at 72°C. Each reaction mixture (25 μl) contained 12.5 μl HotStart Taq Mastermix (Qiagen, GmbH, Hilden, Germany), 1 μl of each primer (2.5 pmol μl^-1^ each, final concentration), 2 μl of bovine serum albumin (10 mg ml^-1^, Amersham Biosciences, Uppsala, Sweden) and 1 μl DNA template. Products were purified with the GenElute PCR Clean-up kit (Sigma, Copenhagen, Denmark), and separated on a 1.5% agrose gel (120V, >60 min). Bands of the correct size (∼830 nucleotides) were excised and cleaned with the GenElute Gel Extraction Kit (Sigma).

### Clone Library Construction, Sequencing and Phylogenetic Tree Reconstruction

Prior to cloning, DNA concentrations of purified, gel-extracted PCR products from individual samples were quantified with a Nanodrop^TM^ spectrophotometer (Thermo Fisher Scientific, Wilmington, Germany). Equal amounts of DNA were pooled to represent replicate sediment cores (individual LF cores) or sampling sites (SKA, BS and M1) by combining PCR products from individual sediment depths. Products from MV sediments and enrichments were not pooled. Cloning was performed using the pGEM-T vector system (Promega Corp., Madison, WI, USA). Plasmids carrying a correct-sized insert were Sanger sequenced (GATC-Biotech, Konstanz, Germany) using the standard vector primer M13F. Sequences (>800 nucleotides) were checked for possible chimeric origin using Bellerophon (Huber et al., 2004), translated into amino acid sequences and manually aligned in ARB (Ludwig et al., 2004). A distance matrix was generated on a total of 805 sequences applying a filter considering 262 amino acid positions that were present in all sequences. Sequences were clustered into operational taxonomic units (OTUs) at 90 and 95% amino acid sequence identity using the furthest neighbor algorithm in mothur_v.1.23.0 (Schloss et al., 2009).

Maximum likelihood trees (RaxML, BLOSUM 62 amino acid substitution model) (Stamatakis, 2006) were calculated in ARB based on translated *masD/assA/bssA/nmsA* gene sequences longer than 1200 nucleotides (>400 amino acids) and applying a 30% amino acid frequency filter. Bootstrap analysis was performed with 100 re-samplings. Shorter reference sequences and partial sequences from this study (one representative sequence per OTU) were added to the tree with the ARB Parsimony interactive tool using the same filter as for tree calculation.

### Statistical Analyses

Principal coordinate analysis (PCoA) and permutational multivariate analysis of variance (PERMANOVA) were used to assess differences between sites and samples, using a cut-off of 95% amino acid sequence identity to define an OTU. PCoA was used for visualization and was based on weighted UniFrac distances. The UniFrac distance matrix was calculated from a RaxML tree that contained representative sequences for OTUs clustered at a cut-off of 95% amino acid sequence identity (see above). PERMANOVA was applied to a Bray–Curtis distance matrix of square-root transformed, relative OTU abundances as well as to the weighted UniFrac distance matrix. PCoA was done in FastUniFrac (Hamady et al., 2009), PERMANOVA in R 2.15.0 (R Development Core Team, 2012) using the vegan package (Oksanen et al., 2007). Diversity and richness estimators (Chao1, Shannon, Inverse Simpson) were calculated with mothur (Schloss et al., 2009). Differences in SCA concentrations between seepage and pristine sampling sites and between sites showing high and low methane concentrations were assessed using Student’s *t*-test. Differences were considered significant at *P*-values <0.05.

### Nucleotide Sequence Accession Numbers

Partial *masD/assA* gene nucleotide sequences are available from the European Nucleotide Archive (ENA) under nucleotide accession numbers LN868278–LN868322.

## Results And Discussion

### Short-Chain Alkane Concentrations in Pristine Sediments

**FIGURE 1:**
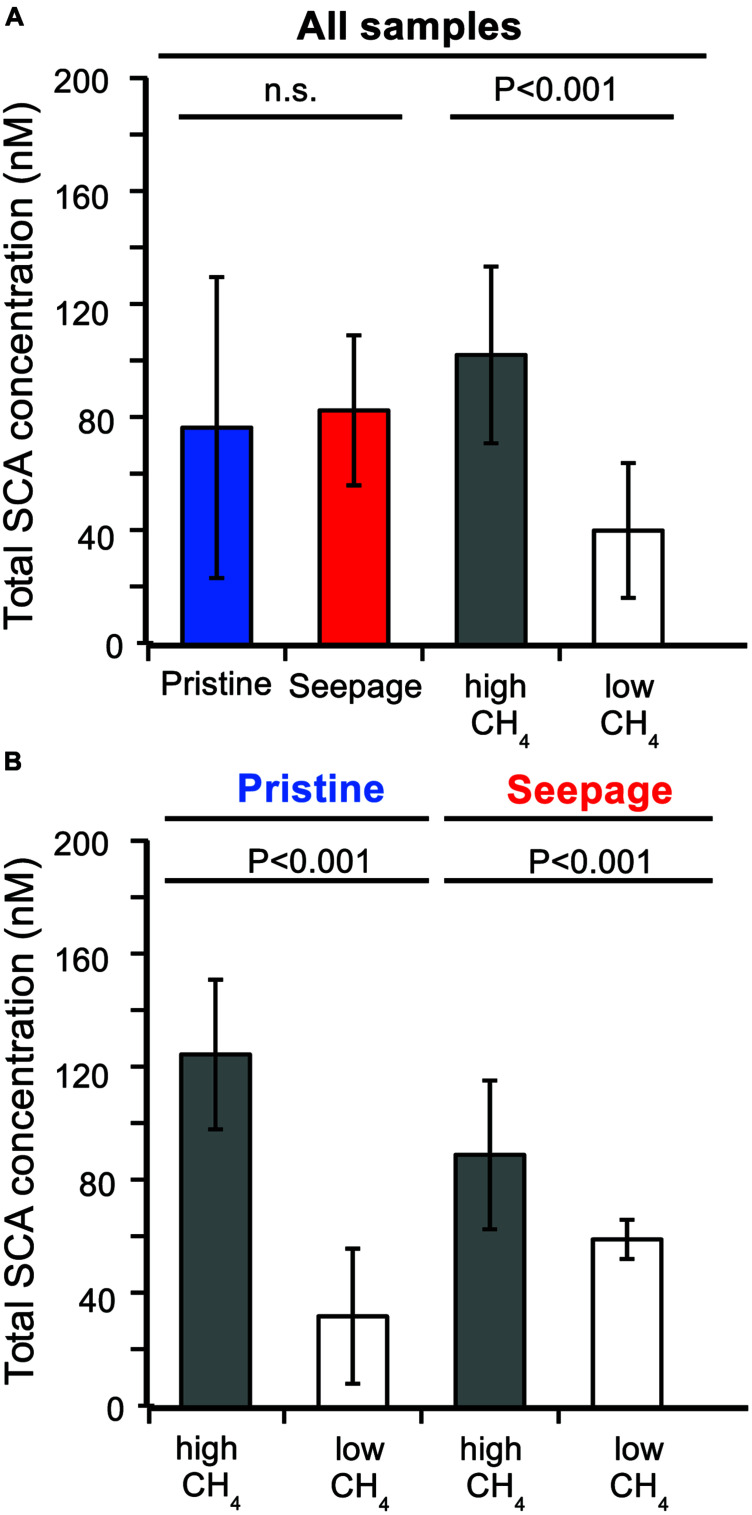
**Average total SCA concentrations in pristine and seepage-impacted sediments. (A)** Comparison based on all samples grouped according to sediment type (pristine vs. seepage) and methane concentrations (high: LF1, LF4, SKA5, M5; low: BB, SKA1, SKA3, SKA4). **(B)** Separate comparisons for pristine and seepage sediments, respectively. *P*-values are indicate based on Student’s *t*-test. n.s., not significant (*P* > 0.05).

**FIGURE 2:**
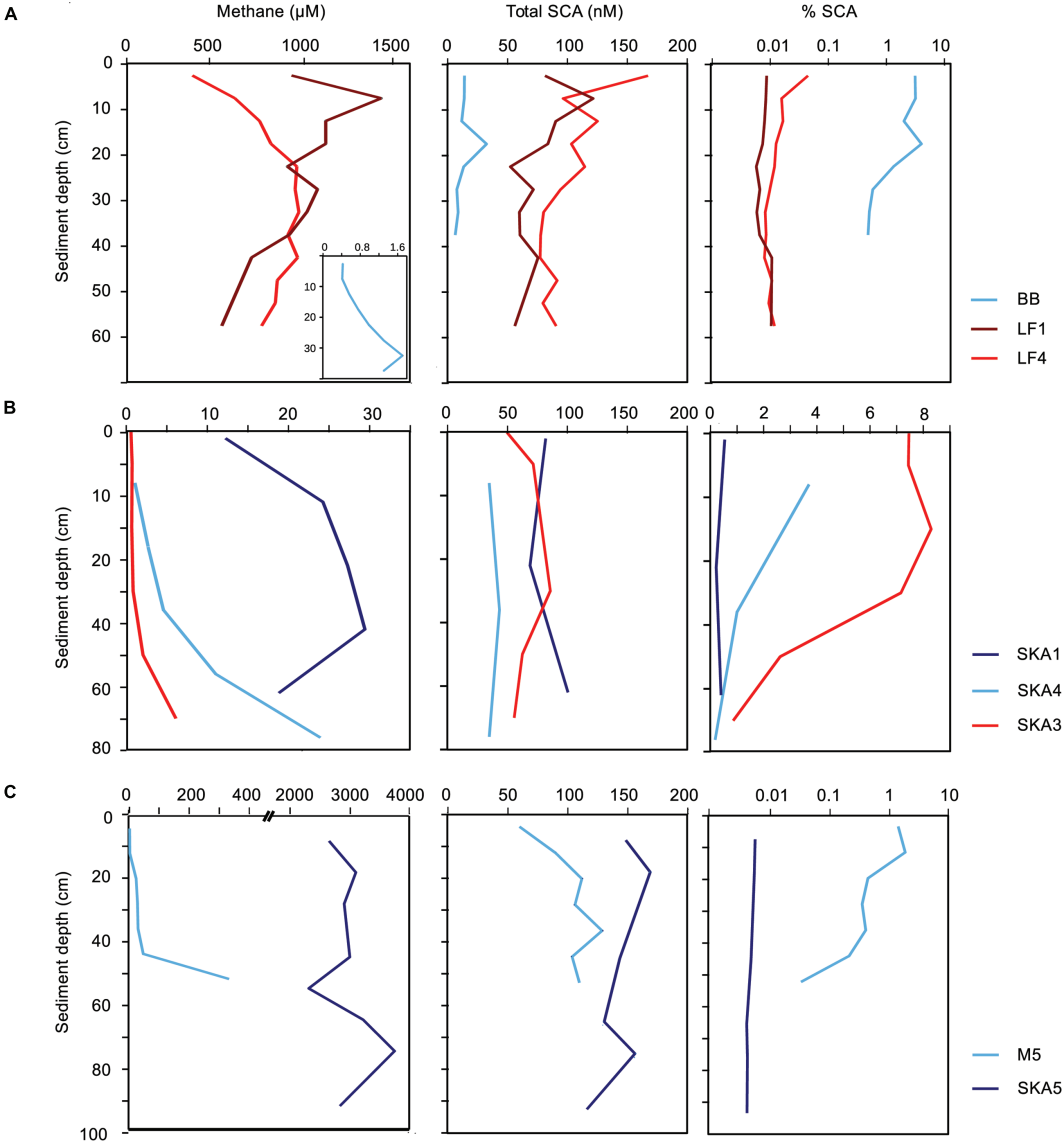
**Methane, total short-chain alkane (SCA) concentrations and their abundance relative to methane in seepage and pristine sediments. (A)** Limfjorden, **(B)** Skagerrak, **(C)** Aarhus Bay (M5) and Lille Belt (SKA5). Red lines: seepage sediments, blue lines: pristine sediments. Please note that methane concentrations and % SCA are displayed on different scales dependent on the sampling site, while total SCA concentrations are displayed on the same scales.

Few studies have reported SCA concentrations for pristine marine sediments, such as shallow and deep subseafloor sediments that are not influenced by the seepage of gas and oil from underlying reservoirs of fossil hydrocarbons. The reported concentrations ranged from few nanomolar to several micromolar (e.g., Kvenvolden and Redden, 1980; Kvenvolden, 1988; Waseda and Didyk, 1995; Kvenvolden and Lorenson, 2000; Hinrichs et al., 2006). In our samples, total SCA concentrations ranged from 10 to 160 nM (**Figures 1** and **2**). On average, pristine sediments showed similar concentrations as seepage-impacted sediments (76 ± 53 and 82 ± 27 nM, respectively, **Figure 1**) and differences were not significant (Student’s *t*-test, *P* > 0.05, **Figure 1A**). However, significant differences were found between samples from methane-rich sediments (SKA5, M5, LF1, LF4) and methane-poor sediments (BB, SKA1, SKA3, SKA4) with average total SCA concentrations of 102 ± 31 nM and 40 ± 24 nM, respectively (Student’s *t*-test, *P* < 0.001, **Figure 1A**). This difference was also significant when comparing samples from pristine and seepage-impacted sediments separately (**Figure 1B**). Seepage sediments in Limfjorden (LF1, LF4, **Figure 2A**) were clearly distinguishable from the nearby pristine reference site (BB) with more than a 1000-fold higher methane concentrations and 8–10 times higher total SCA concentrations. Seepage sediment from the Skagerrak (SKA3) was enriched in SCA relative to methane (% SCA up to 8%, **Figure 2B**) and thereby distinct from the pristine Skagerrak sediments SKA1 and SKA4. Pristine sediments in Aarhus Bay (M5) and Lillebelt (SKA5) were rich in methane and contained relatively high concentrations of total SCA compared to the pristine sediments in Limfjorden and the Skagerrak (**Figure 2C**). In all methane-rich sediments, the carbon isotopic composition for methane pointed to a biogenic source (δ^13^C = 60–74‰). For all methane-poor sediments, the carbon isotopic composition was in a range (δ^13^C = 30–58‰) which, given the low methane concentrations (0.5–30 μM), likely reflects extensive microbial methane oxidation rather than a thermogenic origin of the methane gas.

The nanomolar concentrations of SCA consistently detected in pristine sediments and the observation that SCA concentrations were correlated to the concentration of biogenic methane (**Figure 1**) support the hypothesis that SCA can be formed by biological processes, e.g., by ethanogenesis from ethylene or acetate (Hinrichs et al., 2006; Xie et al., 2013). Our findings thus show that the gas origin (biogenic or thermogenic) cannot be inferred by SCA concentrations and their relative abundance alone, but needs to be verified by, for example, isotopic signatures for the individual components.

### Performance of Optimized PCR Primers in Alkane-Degrading Pure Cultures and Environmental Samples

To comprehensively target known *masD/assA* gene diversity and produce amplification products of a length with sufficient sequence information to allow robust phylogenetic comparisons, we designed and tested four new *masD/assA*-targeted PCR primer pairs (combinations of two newly designed forward and reverse primers, respectively; **Table 2**). These primer pairs covered the gene region that encodes part of the active site of the enzyme including the conserved cysteine residue being diagnostic for MAS/ASS and BSS enzymes. The performance of the different primer pairs was evaluated by PCR amplification of genomic DNA from a selection of sulfate-reducing, alkane-degrading pure cultures (**Table 2**) and subsequent sequencing to verify the identity of the products. Primer pair masD1156a was the only primer combination that successfully amplified *masD/assA* gene fragments (∼830 nucleotides) from all tested pure cultures with no amplification occurring for the negative control culture. Subsequently, performance of the primer pair masD1156a was tested on DNA extracted from environmental samples including pristine and seepage sediments as well as alkane-degrading enrichment cultures (**Table 1**). Amplification was successful for all samples from pristine and seepage sediments and from mesophilic (25°C) enrichment cultures. PCR products consisted of a prominent band of the right-sized fragment (830 nucleotides), but also contained several unspecific products of both longer and shorter fragments. Cloning and sequencing of unspecific products revealed no observable homology with *masD/assA* or *bssA* genes (blastx search). No amplification occurred with DNA extracted from MV enrichment cultures grown under thermophilic conditions (55°C) on either propane (MV55C3) or butane (MV55C4).

**FIGURE 3:**
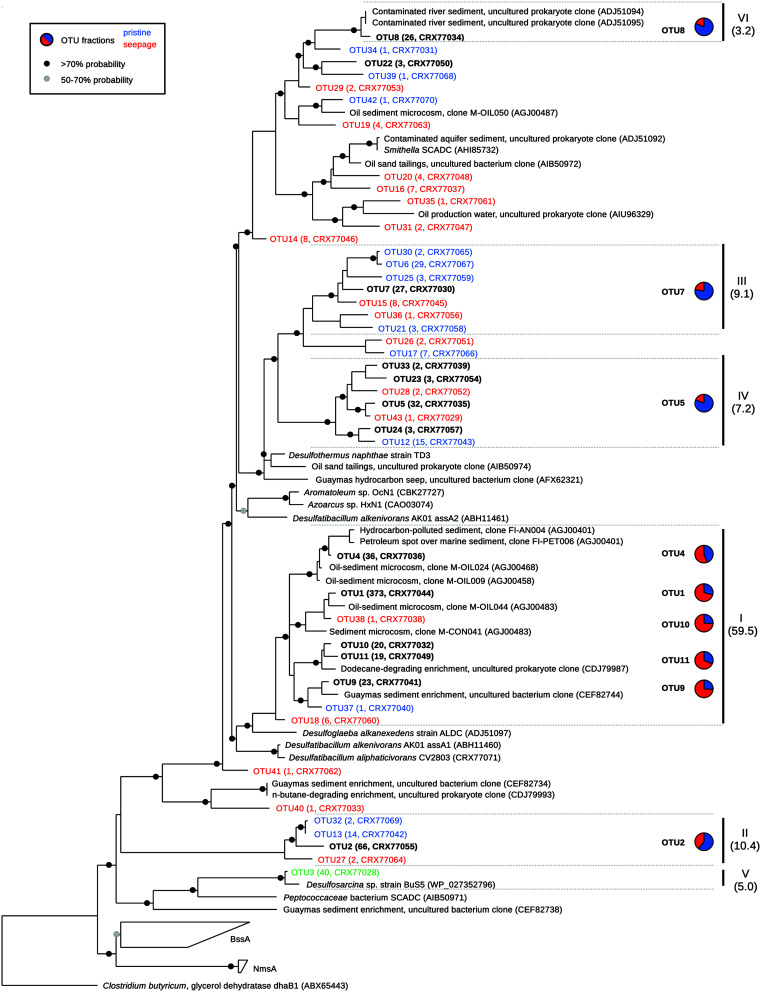
**Phylogeny of partial *MasD/AssA*-like amino acid sequences retrieved from seepage and non-seepage sediments.** Operational taxonomic units (OTUs) were defined at a sequence cut-off of 90% amino acid sequence identity. In red: OTUs recovered only from seepage sites; in blue: OTUs recovered only from pristine sites. OTU3 (green) was exclusively recovered from MV enrichments. In black and bold: OTUs that were present in both seepage and pristine sediments. In parentheses: number of sequences per OTU and protein accession numbers. Pie charts show the relative abundance of sequences per OTU (>3 sequences/OTU) retrieved from seepage (red) and pristine (blue) sediments. Clusters I-VI were defined as sharing 75% amino acid sequence identity (in brackets: relative abundance). Circles on nodes indicate bootstrap values according to the legend.

**FIGURE 4:**
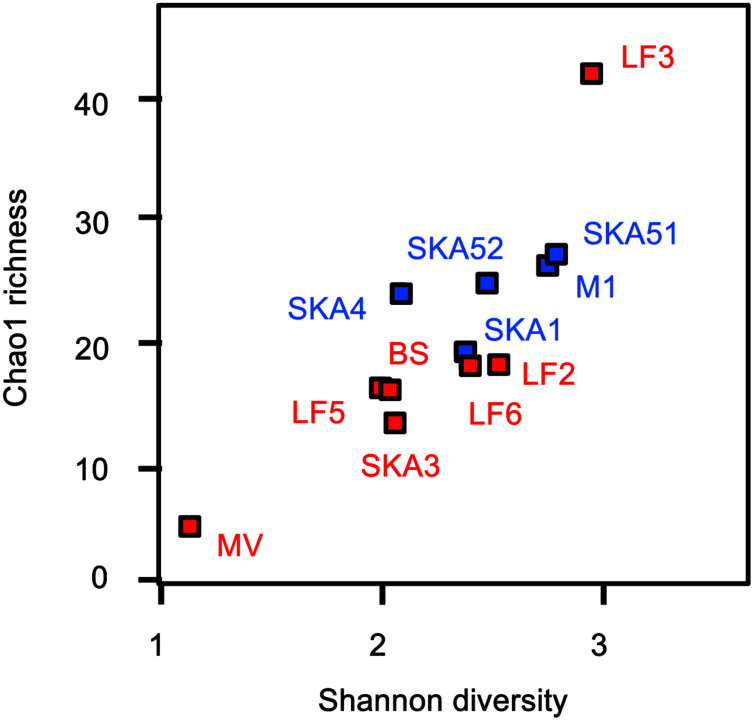
***MasD/assA* gene sequence diversity and richness indices.** Red symbols: seepage sediments, blue symbols: pristine sediments. Indices for MV enrichments are not shown (please refer to Supplementary Table S2).

Clone libraries were constructed from all samples (except MV55C3, MV55C4), yielding a total of 805 high-quality sequences which all represented *masD/assA* genes. Sequences clustered in 43 and 85 OTUs (>90 and >95% amino acid sequence identity, respectively). OTUs were either affiliated with environmental sequences from hydrocarbon-contaminated environments, natural seepage sites and hydrocarbon-degrading enrichments (Callaghan et al., 2010; Acosta-González et al., 2013; Kleindienst et al., 2014; Bian et al., 2015; Tan et al., 2015) or formed novel clades in the *masD/assA* gene phylogeny, likely reflecting the improved primer coverage (**Figure 3**). Out of 43 OTUs (>90% amino acid sequence identity), 17 and 12 OTUs were exclusively recovered from seepage or pristine sediments, respectively, while 14 OTUs were shared, including the most abundant OTUs (**Figure 3**, Supplementary Table S1). *MasD/assA* gene OTU richness (Chao1 estimate) was slightly higher in pristine than in seepage samples, but not statistically significant (**Figure 4**, Supplementary Table S2). Likewise, there was no significant difference in diversity measures (**Figure 4**, Supplementary Table S2). We recovered a much higher *masD/assA* gene phylotype richness in seepage and pristine sediments than reported in previous studies (Kimes et al., 2013; Johnson et al., 2015). For comparison to these studies, we clustered *masD/assA* gene sequences from our study at a cut-off of 97% amino acid sequence identity and identified a total of 134 OTUs with 16–32 OTUs per sampling site. In contrast, only a total of 13 and 18 OTUs were identified in hydrocarbon-impacted sediments from the Gulf of Mexico (8 OTUs per sampling site; Kimes et al., 2013) and Chesapeake Bay (1–5 OTUs per sampling site; Johnson et al., 2015), respectively. This difference can be attributed to environmental factors such as the hydrocarbon load or the geochemistry of the sediment, but more likely reflects the limited coverage of the *masD/assA* gene primers used in previous studies.

Our primers were designed to target all known *masD/assA* genes from anaerobic alkane degraders available in pure culture without any mismatches (Supplementary Table S3) and additionally showed no mismatch wih the *masD/assA* gene recovered from the single cell-amplified genome of *Smithella* sp. SCADC (AHI85732). The pure cultures represent medium- to long-chain alkane degraders with the exception of strain BuS5, the only SCA degrader so far isolated in pure culture (Kniemeyer et al., 2007). The *masD/assA* gene of strain BuS5 has a basal position in the *masD/assA* gene phylogeny and forms a lineage, which is highly underrepresented in public sequence repositories (**Figure 3**). For this reason, we cannot conclusively assess if our primers are likely to amplify the *masD/assA* genes of relatives of strain BuS5.

### Contrasting *masD/assA* Gene Phylotype Composition in Seepage-Impacted and Pristine Sediments

**FIGURE 5:**
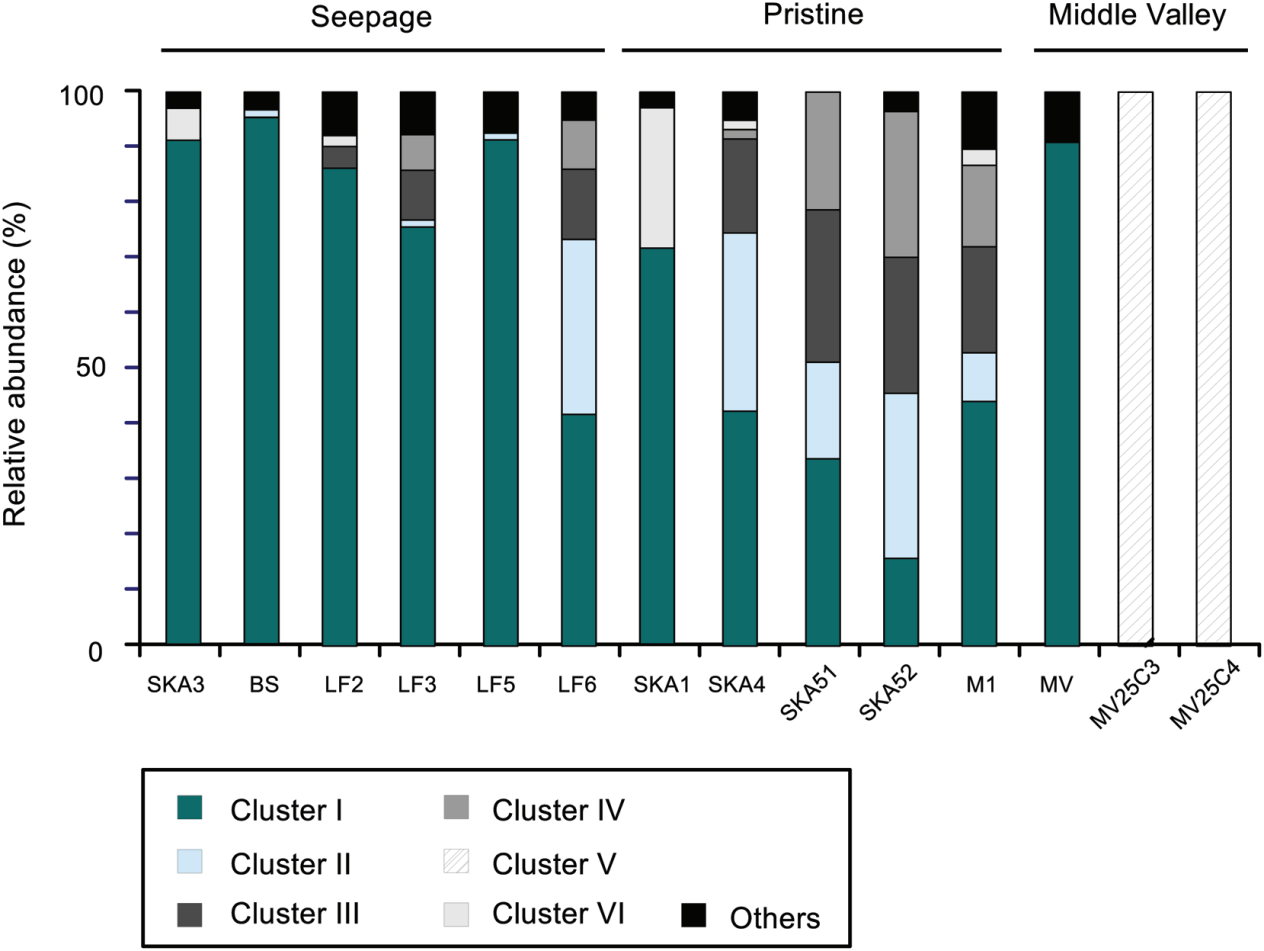
**Relative abundance of the six most abundant *masD/assA* gene sequence clusters.** Clusters I–VI were defined as sharing 75% amino acid sequence identity (see **Figure 1**).

The phylogenetic analysis of *masD/assA* gene clone libraries identified six main clusters each representing >3% of all sequences and collectively accounting for >95% of all sequences retrieved (**Figures 3** and **5**). These clusters grouped sequences that shared more than 75% amino acid sequence identity. Different sequence clusters were predominant in seepage and non-seepage sediments (**Figures 3** and **5**). Seepage samples (except for LF6) were clearly dominated by sequences affiliated with cluster I (77–95% of *masD/assA* gene clone libraries of individual samples). This cluster was also targeted by previously published *masD/assA* gene-targeted primers (Callaghan et al., 2010; von Netzer et al., 2013) and contained sequences retrieved from oil-polluted marine sediments (Acosta-González et al., 2013) and long-chain alkane-degrading enrichment cultures from the Guaymas Basin (Kleindienst et al., 2014), with a distant relationship to *Desulfatibacillum alkenivorans* AK-01 and *Desulfoglaeba alkanexedens* ALDC (77–86% amino acid sequence identity, **Figure 3**). Only 16–44% of all *masD/assA* gene sequences from pristine sediments were affiliated with cluster I, except for SKA1 (73%). In pristine sediments, clusters II, III, IV, and VI were predominant and accounted for up to 32, 25, 26, and 25%, respectively. Cluster II did not include any isolates or environmental sequences from previous studies and thus represented a novel *masD/assA* gene phylotype mainly associated with pristine sediments. Sequences affiliated with clusters III and IV were only distantly related to *Desulfothermus naphthae* TD3 (Rueter et al., 1994) and to environmental sequences retrieved from oil sand tailings (Tan et al., 2015) and hydrocarbon seeps in the Guaymas Basin (von Netzer et al., 2013) sharing 69–79% amino acid sequence identity (**Figure 3**). Cluster VI was represented by a single OTU (OTU8) and closely affiliated (96% amino acid sequence identity) with environmental sequences recovered from contaminated river sediments (Callaghan et al., 2010).

**FIGURE 6:**
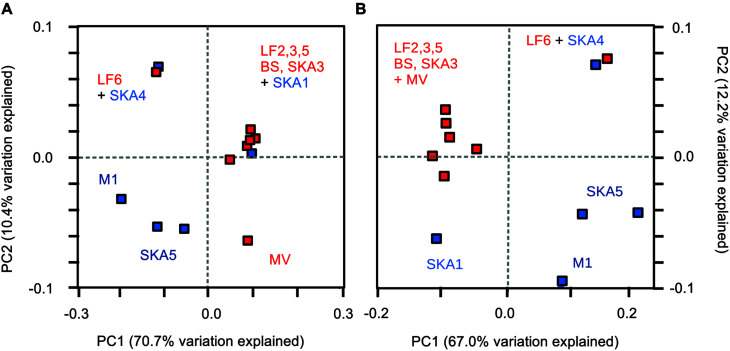
**Phylogenetic dissimilarity between samples as assessed by principle coordinate analysis (PCoA) based on weighted UniFrac distances.** OTUs were defined at a sequence cut-off of 95% amino acid sequence identity, and PCoA analysis was performed including **(A)** all OTUs and **(B)** excluding the two most abundant OTUs from the analysis. Red symbols: seepage sediments, blue symbols: pristine sediments. Sequences from MV enrichments were excluded from the analysis.

Principal coordinate analysis based on weighted UniFrac distances (i.e., considering phylogenetic relationships) was used to visualize clusters of similar communities according to sediment type (**Figure 6**). MV enrichments were excluded from the analysis, as they harbored only a single phylotype and therefore obscured variability patterns. Pristine and seepage sediments were separated along the first principal coordinate (**Figure 6A**). PCoA thus supported the pattern observed from relative cluster abundances and OTU composition (**Figures 3** and **5**). The more similar taxonomic community composition in the majority of seepage sediments (**Figure 5**) was reflected in a tight clustering of these sediments, whereas pristine sediments were more divergent across sites and samples (**Figure 6A**). PERMANOVA (based on weighted UniFrac distances) on all samples showed no statistical differences between sampling sites and sample types [sites: Pseudo-*F* = 0.53, *p*(perm) > 0.8); types: Pseudo-*F* = 0.56, *p*(perm) > 0.5]. Samples from the seepage site LF6 and the pristine site SKA1 did not cluster with their respective sample types. Excluding these two samples and MV from the statistical analysis indeed resulted in a significant difference in *masD/assA* gene phylotype composition between sites [Pseudo-*F* = 8.86, *p*(perm) = 0.036] and sample types [Pseudo-*F* = 11.58, *p*(perm) = 0.012] and supported the tight clustering by sample type. To test the hypothesis that highly abundant OTUs masked a more close affiliation, and thus tighter clustering of pristine sediments, OTU1 and OTU2 (total relative abundance: 24%) were removed from the PCoA analysis (**Figure 6B**). This analysis included MV, but not LF6 into the seepage cluster and separated the pristine sample SKA1 from the seepage sites (**Figure 6B**). The more pronounced separation of pristine and seepage sediments was supported by PERMANOVA on the corresponding weighted UniFrac distance matrix. Significant differences were calculated between sampling sites [Pseudo-*F* = 8.61, *p*(perm) = 0.015] and sediment types [Pseudo-*F* = 8.45, *p*(perm) = 0.006].

### Key Players in SCA Degradation in the Middle Valley Hydrothermal Vent System and Implications for Cold Marine Sediments

In the MV hydrothermal vent system, sediments are characterized by high concentrations of dissolved hydrocarbon species (several 100’s of micromolar of total C_2_–C_4_ alkanes) (Cruse and Seewald, 2006) together with high concentrations of inorganic reduced compounds (H_2_, metals and metal sulfides) and low amounts of organic carbon (0.3–0.5%) supporting the coupling of SCA degradation to sulfate and/or metal reduction (Cruse and Seewald, 2006; Wankel et al., 2012; Adams et al., 2013). Sediments from this system and from the respective batch reactor enrichments (propane- and butane-degrading under sulfate-reducing conditions, 25 and 55°C) (Adams et al., 2013) were thus chosen to compare their *masD/assA* gene phylotype composition to that of pristine and seepage-impacted cold marine sediments with low SCA concentrations. *MasD/assA* gene diversity in MV sediments was very low compared to seepage sites in Limfjorden and the Skagerrak with only 4 OTUs being identified at cut-off levels of 90% and 95% amino acid sequence identity (**Table 1**, Supplementary Table S2). Three of these OTUs were exclusively recovered from MV sediment (OTUs 18, 35, 41) and PCoA analysis (weighted UniFrac distances, all OTUs) pointed to a distinctly different putatively alkane-degrading microbial community than in cold marine sediments from Limfjorden and the Skagerrak (**Figure 6A**).

*MasD/assA* genes were successfully PCR-amplified from mesophilic enrichments (25°C) with propane and butane, while amplification from thermophilic enrichments (55°C) failed with both our novel primer set and previously published primers (von Netzer et al., 2013). Mesophilic enrichments were dominated by a single *masD/assA* gene phylotype (OTU3, **Figure 3**). Sequences shared >98.7% amino acid sequence identity with each other and were closely (95% amino acid sequence identity) related to *Desulfosarcina* sp. strain BuS5. This finding was in line with previous studies that used 16S rRNA-based approaches and that identified strain BuS5 and relatives as important SCA degraders in low temperature environments and under mesophilic enrichment conditions (Kniemeyer et al., 2007; Bose et al., 2013; Jaekel et al., 2013; Kleindienst et al., 2014), whereas they were not detected in thermophilic enrichments (Adams et al., 2013). No *masD/assA* gene sequences closely affiliated with strain BuS5 were found in cold marine sediments in Danish waters. Likely strain BuS5-like bacteria are of very low abundance and instead unknown *masD/assA* gene-carrying microorganisms may be fueled by the nanomolar concentrations of SCA in these sediments, e.g., members of the *masD/assA* gene clusters II, III, and IV. Previous 16S rRNA-based studies on mesophilic SCA-degrading enrichment cultures revealed the presence of deltaproteobacterial sulfate reducers that were distinct from strain BuS5, but closely related to members of the genera *Desulfobacter* and *Desulfobacula* (Savage et al., 2010; Bose et al., 2013; Jaekel et al., 2013). The diversity of mesophilic SCA degraders, and consequently *masD/assA* gene diversity, is clearly greater than previously recognized. Members of the so far unrecognized *masD/assA* gene clusters might just as well represent lineages of anaerobic alkane degraders that utilize medium- to long-chain alkanes, which may be important constituents in marine sediments and can be of both petrogenic and biogenic origin (e.g., from plant waxes, microalgae and cyanobacteria) (Volkman et al., 1992). Therefore, the hypothesis that SCA degraders form a separate *masD/assA* gene lineage distinct from medium- and long-chain alkane degraders (Musat, 2015) and the preference of various clusters for different substrate chain lengths needs to be further investigated, preferentially using approaches that include stable isotope labeling and (meta-) genomic analyses of enrichment cultures.

In thermophilic MV batch reactors, Adams et al. (2013) identified a distinct dominant lineage of *Deltaproteobacteria*, which was most closely related to a thermophilic butane-degrading sulfate reducer from the Guaymas Basin (Kniemeyer et al., 2007) and tentatively identified as member of the family *Desulfurellaceae*. Members of this family were found in surficial Guaymas Basin sediments rich in ANME-1 (Teske et al., 2002) and were proposed to constitute the sulfate-reducing partner in a consortium mediating thermophilic anaerobic oxidation of methane (AOM) (Holler et al., 2011; Kellermann et al., 2012). Possibly, members of this lineage are not only ecologically relevant as partners in thermophilic AOM, but also as SCA degraders at elevated temperatures. It was previously suggested that these *Deltaproteobacteria* are not obligate partners of ANME-1 archaea and may be able to perform sulfate reduction independent from AOM (McKay, 2014). However, the mechanism of alkane activation in thermophilic SCA degraders remains unknown and our results indicated that *masD/assA* genes were either very dissimilar from the known gene diversity and therefore not targeted by our primers or that SCA activation by members of this lineage might not proceed through fumarate addition, but through an alternative mechanism (e.g., carboxylation, oxygen-independent hydroxylation).

## Summary And Perspectives

Our study highlights that anaerobic alkane degraders are ubiquitously present in marine sediments, including sites that are isolated from deep oil and gas formations and that are not exposed to high loads of anthropogenic hydrocarbon inputs. Previous studies either failed to detect *masD/assA* genes at pristine sites with the PCR conditions and primers used (Kimes et al., 2013) or analyzed only a small set of sequences from control sites and/or control incubations (Acosta-González et al., 2013). Their ubiquity and the observation of shared phylotype richness in both pristine and seepage-impacted sites support the hypothesis that marine sediments harbor a seed bank of anaerobic alkane degraders that may proliferate when exposed to elevated alkane concentrations (as a result of, e.g., gas pipeline leakage, oil spills and increased ship traffic). The seed bank hypothesis was previously proven for aerobic hydrocarbon degraders in marine pelagic environments (Harayama et al., 2004) and our findings imply that it is also valid for anaerobic alkane degraders in marine sediments.

Alkane hydroxylases (AH) that catalyze the aerobic degradation of alkanes are very diverse, even within the same species, and primers that aim to comprehensively target this diversity very likely produce false positives (Heiss-Blanquet et al., 2005). AH also catalyze other reactions such as the oxidation of terminal alcohols and fatty acids (van Beilen et al., 2005) and are thus not exclusively indicative for aerobic alkane degradation. In contrast to seawater and hydrocarbon plumes, anoxic sediments represent a confined sample type which allows us to more accurately identify seepage sites based on hydrocarbon gas measurements and anomalies in molecular signatures. Our study shows that *masD/assA* genes are a relevant diagnostic marker for anaerobic alkane degradation in cold marine sediments and we thus propose that the quantification of anaerobic alkane degraders in surficial marine sediments will provide a more reliable tool in bioprospecting for oil and gas than the quantification of aerobic alkane degraders.

## Author Contributions

AG, KK, HR, and BJ designed the research presented in this manuscript. KK, HR, and PG collected samples from pristine and seepage sediments and provided samples from alkane-degrading enrichment cultures. AG and JD performed all analytical analysis including gas analytics, molecular biological work, sequence data and statistical analyses. AG wrote the manuscript with input from KK, HR, PG, and BJ.

## Conflict of Interest Statement

The authors declare that the research was conducted in the absence of any commercial or financial relationships that could be construed as a potential conflict of interest.
