# Supplementary material for: Ubiquitous Presence and Novel Diversity of Anaerobic Alkane Degraders in Cold Marine Sediments
Source: Front Microbiol. 2015 Dec 17;6:1414. doi: 10.3389/fmicb.2015.01414 (PMC4681840; doi:10.3389/fmicb.2015.01414)
Supplement: Supplementary file 1 [file Data_Sheet_1.PDF]

Table S1. Representative *mas*D gene sequences as obtained from clone library analysis of seepage and pristine sediment samples. In red: OTUs exclusively represented by seepage sediments, in blue: OTUs exclusively found in non-seepage sediments. OTUs were defined at a cut-off level of 90% amino acid sequence identity.

OTU	Representative sequence	Number of sequences per OTU	Closest protein match (organism, acc. no., identity) †	Source of closest match
**1**	LF2_001_OTU1	373	alkylsuccinate synthase alpha subunit, partial [uncultured bacterium] (AGJ00483, 92%)	marine sediment microcosm + crude oil
**2**	LF6_060_OTU2	66	hypothetical protein [Peptococcaceae bacterium BRH_c4a] (WP_045636236, 51%)	
**3 ***	MV25C31_001	40	hypothetical protein [Desulfosarcina sp. BuS5] (WP_027352796, 95%)	
**4**	LF5_002_OTU4	36	alkylsuccinate synthase alpha subunit, partial [uncultured bacterium] (AGJ00468, 97%)	marine sediment microcosm + crude oil
**5**	LF3_003_OTU5	32		
**6**	M1_031_OTU6	29	alkylsuccinate synthase [Desulfoglaeba alkanexedens ALDC] (ADJ51097, 76%)	
**7**	LF6_008_OTU7	27	alkylsuccinate synthase [Desulfoglaeba alkanexedens ALDC] (ADJ51097, 76%)	
**8**	LF2_012_OTU8	26	alkylsuccinate synthase, partial [uncultured prokaryote] (ADJ51094, 96%)	contaminated river sediment
**9**	SKA3_053_OTU9	23	hypothetical protein [Desulfatibacillum aliphaticivorans] (WP_028315530, 82%)	
**10**	BS_010_OTU10	20	alkylsuccinate synthase alpha subunit, partial [uncultured bacterium] (AGJ00483, 88%)	marine sediment microcosm + crude oil
**11**	LF2_057_OTU11	19	alkylsuccinate synthase alpha subunit, partial [uncultured bacterium] (AGJ00483, 89%)	marine sediment microcosm + crude oil
**12**	SKA52_005_OTU12	15	AssA [uncultured bacterium] (AIB50974, 78%)	oil sands tailings
**13**	SKA51_015_OTU13	14	hypothetical protein [Smithella] (WP_037380663, 52%)	
**14**	LF2_027_OTU14	8	Alkylsuccinate synthase (I), alpha subunit (AssA1) [Desulfatibacillum alkenivorans AK-01] (ACL03428, 85%)	
**15**	LF2_025_OTU15	8	alkylsuccinate synthase [Desulfoglaeba alkanexedens ALDC] (ADJ51097, 76%)	
**16**	LF5_013_OTU16	7	AssA [uncultured bacterium] (AIB50972, 79%)	oil sands tailings
**17**	M1_021_OTU17	7	hypothetical protein [Desulfatibacillum aliphaticivorans] (WP_028315530, 75%)	
**18****	MV_013_OTU18	6	hypothetical protein [Desulfatibacillum aliphaticivorans] (WP_028315530, 85%)	
**19**	BS_070_OTU19	4	alkylsuccinate synthase alpha subunit, partial [uncultured bacterium] (AGJ00487, 81%)	marine sediment microcosm + crude oil
**20**	LF2_041_OTU20	4	AssA [uncultured bacterium] (AIB50972, 83%)	oil sands tailings
**21**	M1_019_OTU21	3	alkylsuccinate synthase [Desulfoglaeba alkanexedens ALDC] (ADJ51097, 75%)	
**22**	LF3_050_OTU22	3	hypothetical protein [Smithella] (WP_037380663, 75%)	
**23**	LF6_057_OTU23	3	hypothetical protein [Desulfatibacillum aliphaticivorans] (WP_028315530, 78%)	
**24**	LF6_082_OTU24	3	hypothetical protein [Desulfatibacillum aliphaticivorans] (WP_028315530, 78%)	
**25**	M1_038_OTU25	3	alkylsuccinate synthase [Desulfoglaeba alkanexedens ALDC] (ADJ51097, 76%)	
**26**	LF3_072_OTU26	2	AssA [uncultured bacterium] (AIB50974, 79%)	oil sands tailings
**27**	BS_113_OTU27	2	hypothetical protein [Smithella] (WP_037380663, 53%)	
**28**	LF3_076_OTU28	2	AssA [uncultured bacterium] (AIB50974, 79%)	oil sands tailings

**29**	LF3_095_OTU29	2	hypothetical protein [Desulfatibacillum aliphaticivorans] (WP_028315530, 82%)	
**30**	M1_015_OTU30	2	alkylsuccinate synthase [Desulfoglaeba alkanexedens ALDC] (ADJ51097, 67%)	
**31**	LF2_034_OTU31	2	hypothetical protein, partial [Smithella sp. SCADC] (WP_037382242, 74%)	
**32**	M1_096_OTU32	2	hypothetical protein [Smithella] (WP_037380663, 48%)	
**33**	LF6_013_OTU33	2	hypothetical protein [Desulfatibacillum aliphaticivorans] (WP_028315530, 77%)	
**34**	SKA1_012_OTU34	1	Alkylsuccinate synthase (I), alpha subunit (AssA1) [Desulfatibacillum alkenivorans AK-01] (ACL03428, 80%)	
**35**	MV_017_OTU35	1	alkylsuccinate synthase [Desulfoglaeba alkanexedens ALDC] (ADJ51097, 76%)	
**36**	LF6_080_OTU36	1	alkylsuccinate synthase [Desulfoglaeba alkanexedens ALDC] (ADJ51097, 75%)	
**37**	SKA1_031_OTU37	1	alkylsuccinate synthase alpha subunit, partial [uncultured bacterium] (AGJ00401, 90%)	hydrocarbon polluted marine sediments
**38**	LF5_024_OTU38	1	alkylsuccinate synthase alpha subunit, partial [uncultured bacterium] (AGJ00483, 86%)	marine sediment microcosm + crude oil
**39**	M1_094_OTU39	1	AssA [uncultured bacterium] (AIB50974, 77%)	oil sand tailings
**40**	BS_013_OTU40	1	(1-methylalkyl)succinate synthase, partial [uncultured bacterium] (CDJ79993, 79%)	anoxic sediment enrichment (n-butane + sulfate)
**41**	MV_039_OTU41	1	hypothetical protein [Desulfatibacillum aliphaticivorans] (WP_028315530, 89%)	
**42**	SKA4_112_OTU42	1	alkylsuccinate synthase alpha subunit, partial [uncultured bacterium] (AGJ00487, 87%)	marine sediment microcosm + crude oil
**43**	LF3_007_OTU43	1	AssA [uncultured bacterium] (AIB50974, 76%)	oil sand tailings

* OTU3 was exclusively represented by clones retrieved from propane- and butane-degrading mesophilic enrichment cultures from a seepage sediment (Middle Valley).
** OTU18 was exclusively represented by clones retrieved from Middle Valley sediment.
† BLAST: May 21st 2015

Table S2. Richness and diversity estimates for *masD* gene clone libraries from pristine and seepage-impacted environmental samples and enrichment cultures.

	Sample	Chao1 richness estimate [b]	Shannon Index [b]	Inverse Simpson Index [b]
**Pristine sediments**				
	M1	26.7 (23.0, 44.8)	2.7 (2.5, 3.0)	12.9 (9.1, 22.1)
	SKA1	19.5 (17.4, 34.0)	2.4 (2.1, 2.6)	7.4 (5.1, 13.2)
	SKA4	24.3 (16.9, 60.0)	2.1 (1.8, 2.4)	5.7 (4.2, 8.9)
	SKA51	27.6 (23.2, 49.1)	2.8 (2.6, 2.9)	15.3 (12.3, 20.4)
	SKA52	25.2 (19.6, 50.4)	2.5 (2.2, 2.7)	10.0 (7.4, 15.6)
**Seepage-impacted sediments**				
	LF2 [b]	18.4 (17.2, 27.6)	2.5 (2.3, 2.8)	10.7 (7.3, 20.3)
	LF3 [b]	42.6 (31.2, 84.5)	2.9 (2.7, 3.1)	16.4 (12.0, 25.8)
	LF5 [b]	16.5 (15.2, 27.5)	2.0 (1.7, 2.2)	4.3 (3.2, 6.8)
	LF6 [b]	18.3 (18.0, 22.7)	2.4 (2.1, 2.6)	7.4 (5.3, 12.2)
	SKA3	13.6 (13.1, 20.1)	2.0 (1.8, 2.3)	5.8 (4.4, 8.5)
	BS	16.3 (13.5, 35.1)	2.0 (1.8, 2.3)	5.7 (4.2, 8.8)
	MV	5.0 (4.1, 17.3)	1.1 (0.7, 1.6)	3.1 (1.8, 8.8)

[a] Defined at a cutoff of 5% amino acid sequence difference. Lower and higher bounds for 95% confidence intervals are given in brackets.
[b] 4 cores (LF2, LF3, LF5 and LF6) were taken in the seepage-impacted area and analyzed separately.

Table S3. Primer sequences and corresponding positions of aligned nucleotide sequences of tested pure cultures and other representatives (*Smithella* sp. SCADC, *Peptococcaceae* bacterium SCADC).

		**forward primer masD1156F**						**reverse primer masD2004Rmod**						
Nucleotide sequence (5'-3')		**GGH**	**MCV**	**TDB**	**GTV**	**TGG**	**AC**	RTC	RTC	RTT	DCC	CCA	YTT	NGG
Nucleotide sequence (reverse complement)								**CCN**	**AAR**	**TGG**	**GGH**	**AAY**	**GAY**	**GAY**
Tested pure cultures														
Azoarcus sp. HxN1	(CAO03074)	GG**T**	**A**C**G**	T**TC**	GT**G**	TGG	AC	CC**G**	AA**A**	TGG	GG**T**	AA**T**	GA**T**	GA**T**
Aromatoleum sp. OcN1	(CBK27727)	GG**T**	**A**C**G**	T**TC**	GT**G**	TGG	AC	CC**G**	AA**A**	TGG	GG**C**	AA**T**	GA**T**	GA**C**
Desulfatibacillum alkenivorans AK-01 assA1	(ABH11460)	GG**C**	**A**C**C**	T**GG**	GT**C**	TGG	AC	CC**C**	AA**A**	TGG	GG**C**	AA**T**	GA**C**	GA**C**
Desulfatibacillum alkenivorans AK-01 assA2	(ABH11461)	GG**C**	**A**C**C**	T**GG**	GT**G**	TGG	AC	CC**C**	AA**A**	TGG	GG**C**	AA**C**	GA**C**	GA**C**
Desulfatibacillum alkenivorans PF2803^T	(CRX77072)	GG**C**	**A**C**C**	T**GG**	GT**C**	TGG	AC	CC**C**	AA**A**	TGG	GG**C**	AA**T**	GA**C**	GA**C**
Desulfatibacillum aliphaticivorans CV2803^T	(CRX77071)	GG**C**	**A**C**C**	T**GG**	GT**C**	TGG	AC	CC**C**	AA**A**	TGG	GG**C**	AA**T**	GA**C**	GA**C**
Desulfoglaeba alkanexedens ALDC^T	(ADJ51097)	GG**C**	**A**C**A**	T**GG**	GT**C**	TGG	AC	CC**C**	AA**G**	TGG	GG**A**	AA**T**	GA**T**	GA**C**
Desulfothermus naphthae TD3^T	(unpublished)	GG**T**	**A**C**A**	T**GG**	GT**C**	TGG	AC	CC**T**	AA**A**	TGG	GG**C**	AA**T**	GA**T**	GA**T**
Desulfosarcina sp. strain BuS5	(WP_027352796)	GG**C**	**C**C**G**	T**AT**	GT**A**	TGG	AC	CC**T**	AA**G**	TGG	GG**T**	AA**T**	GA**T**	GA**T**
Other representatives														
Smithella sp. SCADC	(AHI85732)	GG**A**	**A**C**A**	T**GG**	GT**C**	TGG	AC	CC**T**	AA**A**	TGG	GG**C**	AA**T**	GA**T**	GA**T**

IUPAC nucleotide code:

R (A/G), Y (C/T), M (A/C), B (C/G/T), D (A/G/T), H (A/C/T), V (A/C/G), N (any base)
